# Low Tube Voltage Liver MDCT with Sinogram-Affirmed Iterative Reconstructions for the Detection of Hepatocellular Carcinoma

**DOI:** 10.1038/s41598-017-10095-6

**Published:** 2017-08-25

**Authors:** B. Pregler, L. P. Beyer, A. Teufel, C. Niessen, C. Stroszczynski, H. Brodoefel, P. Wiggermann

**Affiliations:** 1 Department of Radiology, University Hospital Regensburg, Regensburg, Germany; 2 Institute of Radiology, Hospital Ortenau Lahr-Ettenheim, Lahr, Germany; 3 Department of Internal Medicine I, University Hospital Regensburg, Regensburg, Germany

## Abstract

Aim of this study was to compare low tube voltage computed tomography (80 kV) of the liver using iterative image reconstruction (SAFIRE) with standard computed tomography (120 kV) using filtered back-projection (FBP) for the detection of hepatocellular carcinoma (HCC). 46 patients (43 men) with 93 HCC confirmed by 3 T MRI with Gd-EOB-DPTA, in inconclusive cases combined with contrast-enhanced ultrasound, underwent dual-energy CT. The raw data of the 80 kV tube was reconstructed using the iterative reconstruction algorithm SAFIRE with two strengths (I3 and I5). The virtual 120 kV image data set was reconstructed using FBP. The CT images were reviewed to determine the lesion-to-liver contrast (LLC), the lesion contrast-to-noise ratio (CNR) and the sensitivity. The LLC (57.1/54.3 [I3/I5] vs. 34.9 [FBP]; p ≤ 0.01), CNR (3.67/4.45 [I3/I5] vs. 2.48 [FBP]; p < 0.01) and sensitivity (91.4%/88.2% [I3/I5] vs. 72.0% [FBP]; p ≤ 0.01) were significantly higher in the low-voltage protocol using SAFIRE. Therefore, low tube voltage CT using SAFIRE results in an increased lesion-to-liver contrast as well as an improved lesion contrast-to-noise ratio compared to FBP at 120 kV which results in a higher sensitivity for the detection of HCC.

## Introduction

Contrast-enhanced imaging plays a central role in the diagnosis of hepatocellular carcinoma (HCC) and is used both for follow-up and for early detection in high-risk patients. Arterial hypervascularization with characteristic contrast agent washout is considered to be sufficient evidence of an HCC^1–3^. If a definite diagnosis cannot be made in contrast-enhanced CT or MRI, a second dynamic study is recommended^4^. 3-Tesla (3T) MRI with liver-specific contrast agent (Gd-EOB-DPTA) is currently considered the imaging modality with the highest sensitivity (86%) and PPV (94%) in clinical routine for detection and characterization of HCC^5^.

To diagnose HCC on computed tomography, the maximum possible contrast difference between the HCC lesion and the surrounding liver parenchyma (lesion-to-liver contrast (LLC)) should be targeted. A promising approach to improved detection of HCC in computed tomography is the use of low tube voltage (80 kV) since the mean photon energy of 47 keV to 56 keV is close to the absorption maximum of iodine (33.2 keV)^6^. Therefore, significantly higher levels of contrast can be achieved when using iodine-containing contrast agent at a low tube voltage than at a high tube voltage^7, 8^.

Although the theoretical advantages of low tube voltage protocols have long been known, they are partially offset by an increased image noise which has prevented the routine use of low tube voltage in clinical imaging. Two technical innovations have recently allowed to compensate the loss of image quality: the technical ability of modern X-ray tubes to provide a stable high tube current even at low voltage and the use of iterative reconstruction algorithms, such as SAFIRE^9, 10^.

We postulated that a combination of a low tube voltage protocol and an iterative reconstruction algorithm could result in an increase of LLC of HCC compared to the common 120 kV protocol in combination with filtered back-projection (FBP), the standard reconstruction method. The goal of this study was to compare low tube voltage computed tomography (80 kV) using iterative reconstruction (SAFIRE) to a routine CT protocol with 120 kV and image data reconstruction via FBP. 3T MRI using Gd-EOB-DPTA, in inconclusive cases combined with contrast-enhanced ultrasound, served as the gold standard^11^.

## Materials and Methods

### Study design and patient characteristics

The prospective single-center study was approved by the Ethics Committee of the University Regensburg (approval number 14-101-0325) and carried out in accordance with the relevant guidelines and regulations.

All patients with confirmed HCC who were examined at our institute between March 2015 and April 2016 via computed tomography were included. Written consent for participation in the study as well as publication in anonymized form was obtained from all patients. Exclusion criteria were diffuse HCC and contraindications for MRI examination.

If 3T MRI with the liver-specific contrast agent gadolinium-ethoxybenzyl diethylenetriamine pentaacetic acid (Gd-EOB-DTPA, Primovist®; Bayer Schering Pharma, Berlin, Germany) was not available or older than 14 days at the day of the CT scan, a MRI examination performed as part of the study protocol was added. The 3T MRI were evaluated in consensus by two experienced abdominal radiologists according to LI-RADS criteria^12^. In the cases with inconclusive image findings additional contrast-enhanced ultrasound or biopsy was performed for lesions having high probability of being HCC (LR-4) as part of our clinical routine. Following our study goal and to avoid any ambiguities regarding our defined gold standard we only included hypervascular HCC lesions.

In total, 46 patients with 93 arterial hyperenhancing HCC lesions were able to be included in the study. See Table 1 for the basic characteristics.

**Table 1:** Basic characteristics of patients and HCC lesions.

Patient characteristics	
Female gender – number (%)	3 (6.5%)
Male gender – number (%)	43 (93.5%)
Age - years	Average: 63; range: 44–88
Child A cirrhosis – number (%)	21 (45.7%)
Child B cirrhosis – number (%)	11 (23.9%)
Child C cirrhosis – number (%)	4 (8.7%)
No cirrhosis – number (%)	10 (21.7%)
HCC lesion characteristics	
Median number of HCC per patient	1
Maximum number of HCC per patient	6
Diameter, median (min.-max.) - mm	22 (7–93)
Total number of large HCC lesions (≥3 cm)	21
Total number of small HCC lesion (<3 cm)	72

### CT acquisition

All patients were examined on a dual-source CT scanner (SOMATOM Definition® Flash, Siemens Healthcare, Forchheim, Germany). The intravenous injection of 120 ml of contrast agent (Accupaque 350, GE Healthcare Buchler, Braunschweig, Germany) was performed with a flow rate of 4 ml/s followed by an injection of 40 ml of NaCl. Acquisition in the arterial phase was performed via bolus tracking (CARE Bolus, Siemens Healthcare, Forchheim, Germany) in the aorta with a threshold of 100 HU and a delay of 17 seconds.

All CT scans were acquired in dual-energy mode with tube voltages of 80 kV and 140 kV and the settings specified in Table 2. To reduce the radiation dose, the manufacturer’s own automatic tube current modulation (CareDOSE 4D, Siemens Medical Solutions, Forchheim, Germany) was activated.

**Table 2:** Technical settings for acquiring raw CT data.

Collimation	14 × 1.2 mm
Rotation time	0.5 s
Pitch	0.6
Tube voltage	80 kV: A tube; 140 kV: B tube
Reference current strength	550 mAs: A tube; 213 mAs: B tube

### CT image reconstruction

Iterative reconstruction of the 80 kV raw data was performed using the SAFIRE algorithm (Siemens Healthcare, Forchheim, Germany) at a medium and high setting (I3 and I5) and a soft tissue kernel (I31f). A virtual data set with 120 kV, 70% of which was comprised of the data from the 140 kV tube and 30% of the data from the 80 kV tube, was calculated from the raw data for the two tubes. Virtual 120 kV data was reconstructed using filtered back-projection (FBP) and a soft tissue kernel (B30f). Both image data sets were generated with a slice thickness of 3 mm and an increment of 3 mm.

### MRI acquisition

All MRI scans were acquired within a defined time frame (<2 weeks before/after CT examination) on a 3T MRI scanner (Magnetom Skyra, Siemens Healthcare) with a combination of body and spine coil elements for signal reception. T1-weighted VIBE (volumetric interpolated breath-hold examination) sequences with fat suppression (repetition time (TR) 3.09 ms; echo time (TE) 1.16 ms; flip angle 9°; number of excitations 1; slices 64; reconstructed voxel size 1.3 × 1.3 × 3.0 mm; measured voxel size 1.7 × 1.3 × 4.5 mm) over the entire liver were performed prior to contrast administration and up to 20 minutes after contrast administration. Every sequence was generated during breath-hold and without further system adjustment after contrast administration.

Every patient received a quantity of Primovist as a bolus via a peripheral venous access adapted to the patient’s body size and weight (0.025 mmol/kg body weight) at a flow rate of 1 ml/s. The access was purged with 20 ml of NaCl.

### CEUS acquisition

In inconclusive cases, additional CEUS was performed according to our clinical routine pattern. All patients were examined by an experienced radiologist with national DEGUM stage III using a multi-frequency probe (1–5 MHz, LOGIQ E9, GE). Dynamic CEUS was performed with a bolus injection of 2.4 ml highly echogenic sulfur hexafluoride microbubbles (SonoVue®, Bracco, Milan, Italy) followed by 10 ml of a 0.9% saline bolus. Continuous scanning was conducted for 3 minutes starting immediately after injection to approve or disconfirm the suspected HCC lesions.

### Quantitative image analysis

CT scans were evaluated quantitatively by the study coordinator. To quantify the image data, predefined circular regions of interest (ROI) were placed in the following locations: in the subcutaneous fat tissue, in the liver parenchyma, in the aorta at the level of the coeliac trunc, and in the largest HCC lesion at the maximum diameter. The shape and size of the ROI were constant for all measurements.

For the objective evaluation of image quality and visibility of the HCC, the following parameters were recorded:

- The lesion-to-liver contrast (LLC) was defined as the difference between the mean density of the HCC lesion and mean density of the surrounding liver parenchyma (LLC = HU_Lesion_ − HU_Liver_).
- The lesion contrast-to-noise ratio (CNR) was defined as the quotient of the lesion-to-liver contrast divided by the image noise (determined by the standard deviation of the density values within a ROI in the subcutaneous fat tissue [SD_Fat tissue_]): CNR = LLC/SD_Fat tissue_.

### Qualitative image analysis

The CT scans were evaluated subjectively by two radiologists with abdominal imaging experience. The images were analyzed on two high-resolution monitors used in the clinical diagnostic routine (RX 220 EIZO, Ishikawa, Japan) in a defined soft-tissue window (width: 350 HU, center: 50 HU). The differently reconstructed CT data sets (80 kV IR3, 80 kV IR5, 120 kV FBP) were evaluated in three separate sessions at an interval of at least 4 weeks to minimize a recall bias. All patient-specific data were masked.

The evaluators independently documented the following parameters for all examinations and then came to a consensus decision:

- Location and number of HCC lesions
- Confidence level regarding the maximum number of HCC lesions (1 very confident; 2 somewhat confident; 3 not confident)
- Confidence level that at least one HCC lesion is present in the particular case (1 very confident; 2 somewhat confident; 3 not confident)
- Score to evaluate image quality based on the clinical assessability of the liver artery (1 excellent quality; 2 good quality; 3 moderate quality; 4 non-diagnostic quality)

### Statistical analysis

The JMP statistics software package (SAS Institute, Cary, NC, USA) was used for all statistical calculations. A p-value of ≤0.05 was considered statistically significant. McNemar’s test was used to compare sensitivity at different image reconstruction and tube levels. Chi-squared test was employed to compare sensitivity between small and large HCC lesions for each reconstruction. Not normally distributed data were compared using the Mann-Whitney U-test and normally distributed data using the t-test.

### Data Availability

The datasets generated during and/or analysed during the current study are available from the corresponding author on reasonable request.

## Results

### Quantitative image analysis

Table 3 shows the objective image parameters for every reconstruction in the arterial phase. The average image noise (SD_Fat tissue_) is significantly decreased in the iterative reconstruction I5 at 80 kV compared to FBP with 120 kV. In contrast, no significant difference was seen between the iterative reconstruction I3 at 80 kV and FBP with 120 kV (p = 0.17).

**Table 3:** Objectively determined parameters for evaluating image quality and ability to detect HCC lesions.

	120 kV FBP	80 kV I3	80 kV I5
Image noise (SD_Fat tissue_)			
Mean value	15.2	16.2	12.6
Standard deviation	4.2	2.8	2.7
p-value		0.17	<0.01
Lesion-to-liver contrast (LLC)			
Mean value	34.9	57.1	54.3
Standard deviation	24.0	41.7	41.0
p-value		<0.01	<0.01
Lesion contrast-to-noise ratio (CNR)			
Mean value	2.48	3.67	4.45
Standard deviation	1.99	2.87	3.53
p-value		<0.01	<0.01

**Figure 1:**
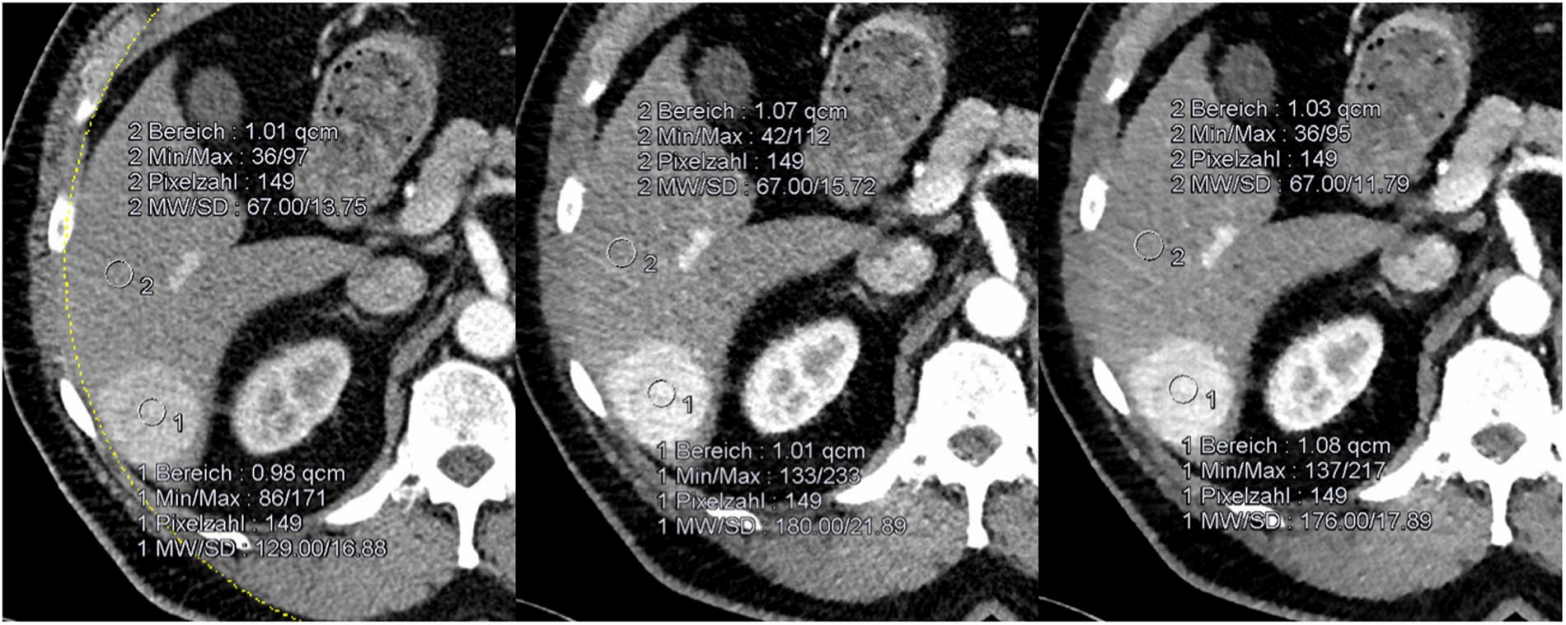
Large HCC in liver segment VI. The lesion contrast-to-noise ratio is significantly higher using SAFIRE than in FBP (4.6 at 120 kV; 7.2 at 80 kV IR3 and 9.2 at 80 kV IR5).

The lesion-to-liver contrast (LLC) and the lesion contrast-to-noise ratio (CNR) were significantly higher at 80 kV with iterative reconstructions I3 and I5 than at 120 kV with filtered back-projection (Fig. 1).

### Qualitative image analysis

The sensitivity for the detection of HCC lesions was significantly higher at 80 kV with iterative reconstruction using SAFIRE (I3 and I5) than at 120 kV with standard FBP. The highest sensitivity (91.4%) was achieved using iterative reconstruction strength 3 (I3) (Table 4).

**Table 4:** Key figures regarding the detection of HCC as a function of tube voltage and image reconstruction method.

	120 kV FBP	80 kV I3	80 kV I5
Confidence max. number of HCC	2.13	1.46	1.46
p-value		<0.001	<0.001
Confidence at least one HCC	1.39	1.06	1.11
p-value		0.001	0.004
True positive - number	67	85	82
False positive - number	3	4	4
False negative - number	26	8	11
Sensitivity (%)	72.0%	91.4%	88.2%
p-value		<0.01	<0.01
Positive predictive value (%)	95.7%	95.5%	95.3%

Two transient hepatic attenuation differences (THAD) and two small arterioportal shunts were falsely classified as HCC using the low tube voltage protocol at both reconstruction strengths (I3 and I5).

The confidence level of the readers in the evaluation of the maximum number of HCC lesions and regarding the presence of at least one HCC lesion was significantly increased at a low tube voltage with iterative image reconstruction compared to 120 kV with FBP.

The sensitivity for large HCC lesions was significantly higher only for 120 kV with standard FBP. A 100% sensitivity was reached for large lesions using 80 kV with iterative image reconstruction (I3 and I5) (Table 5).

**Table 5:** Differences between sensitivity for detection of small and large HCC as a function of tube voltage and image reconstruction method.

Sensitivity	120 kV FBP	80 kV I3	80 kV I5
Small lesions (<3 cm)	66.6%	88.9%	84.7%
Large lesions (≥3 cm)	90.4%	100.0%	100.0%
p-value	0.049	0.186	0.100

The subjective image quality based on the clinical assessability of the liver artery was significantly lower using 80 kV with iterative reconstruction for both IR3 (2.26) and IR5 (3.04) compared to 120 kV with FBP (1.37; p < 0.01).

## Discussion

The theoretical advantage of a low tube voltage when acquiring contrast-enhanced CT scans is known and can be attributed to the low absorption maximum of iodine (32 kV).

Due to two technical innovations, a trend toward a low tube voltage has recently been observed: First, modern CT X-ray tubes can now guarantee a stable tube current even at a low voltage^9^. Second, new iterative reconstruction algorithms (at least partially) offset the negative effect of the increase in noise^13^.

The goal of our study was therefore to compare a combination of low tube voltage (80 kV) and SAFIRE reconstruction to the conventional CT protocol with 120 kV and FBP for the detection of HCC.

Lowering of the tube voltage in computed tomography while maintaining the same current strength results in a significant increase in image noise. Therefore, it was shown, for example, in an earlier study that the image noise was significantly increased in abdominal CT examinations with a low tube voltage (90 kV) compared to traditional CT examinations with 120 kV. Although the level of contrast measured in the aorta increased significantly as a result of the lowering of the tube voltage, the image noise also increased significantly so that the contrast-to-noise ratio became less favorable overall^8^.

The effect of the increase in image noise and the associated decrease in the contrast-to-noise ratio can, at least partially, be minimized or offset by new iterative reconstruction algorithms^10^. A number of studies were able to show that image noise can be reduced and the contrast-to-noise ratio can be increased in low-dose protocols compared to standard protocols by using SAFIRE^14–16^. Data reconstruction using SAFIRE is finding greater clinical use either to increase image quality at a constant dose or to maintain image quality at a lower dose^17, 18^.

In our study, the overall image quality was worse-rated in the 80 kV data set compared to the 120 kV images despite the fact that lowering the tube voltage and using SAFIRE maintained a comparable image noise (I3) or led to an even reduced image noise (I5). This could be referred to the cause that iterative reconstruction algorithms lead to an unfamiliar image appearance, in our case to a “smoothed” impression of the liver artery. Earlier studies pointed out the subjectively poorer graded image quality using iterative image reconstruction methods in spite of the lowered image noise^19^. An increasing implementation of the iterative reconstruction algorithms in the clinical routine should effect a growing familiarization as well as no losses in diagnostic assessability which should be evaluated in future studies.

A study by Schindera *et al*.^10^ demonstrated on the basis of a phantom with hypovascularized liver lesions that lowering of the tube voltage to 100 kV combined with iterative image reconstruction results in a reduction in image noise with an improved contrast-to-nose ratio. When using a low tube voltage protocol with iterative reconstruction, the sensitivity with respect to the detection of lesions on the phantom was comparable to that of the standard protocol with FBP.

A significant improvement in contrast due to lowering of the tube voltage was also able to be achieved in our study as shown by the significant increase in the contrast-to-noise ratio of the aorta in the iterative reconstructions at 80 kV.

The clear contrast enhancement achieved by lowering the tube voltage seems to be a promising approach particularly in the diagnosis of hypervascularized tumors such as HCC. This seems to be of enormous importance especially in patients with liver cirrhosis where inhomogeneous liver texture and multiple dysplastic nodules often aggravate diagnostic analysis in all imaging modalities.

The first study examining the effect of the lowering of the tube voltage (from 140 kV to 80 kV) on the detection of hypervascularized liver tumors was published in 2009. Iterative reconstruction algorithms were not yet available at that time and were therefore not used. Despite a significant increase in image noise and a resulting decrease in the subjectively evaluated image quality, lowering of the tube voltage resulted in an increase in the lesion contrast-to-noise ratio and in improved lesion detection^20^.

In a later study, Marin *et al*.^21^ examined the effect of the newly available iterative image reconstruction algorithm ASiR (adaptive statistical iterative reconstruction; GE Healthcare, Milwaukee, USA) in combination with a low tube voltage on the diagnosis of hypervascularized liver tumors (malignant and benign). As in the first study, a significantly higher lesion contrast-to-noise ratio and improved subjective lesion detection were seen. However, the diagnostic accuracy and the sensitivity with respect to the detection of lesions could not be improved. At the same time, there was an increase in the number of false-positive results although the difference was not statistically significant.

Both studies include a very heterogeneous patient population. Therefore, not only patients with HCC but also patients with other malignant and benign hypervascularized liver tumors such as metastases of renal cell carcinomas and hemangiomas were included. In contrast, our study was limited to patients with hyperenhancing HCC confirmed by imaging. In addition, our study used the new iterative reconstruction algorithm SAFIRE, which, in contrast to ASiR which has already been available for a while, uses a combination of iterations of raw data (sinogram) and image data^22^.

The effect of SAFIRE on the detection of HCC in low-voltage computed tomography was examined by Yu *et al*.^23^. In contrast to our study, only low-voltage CT scans at a tube voltage of 80 kV were acquired in this study. SAFIRE reconstructions with a half tube current (300 mAs) were compared to FBP reconstructions with a full tube current (600 mAs). The authors concluded that comparable diagnostic accuracy to that of image reconstruction with FBP at full dose can be achieved with image reconstruction using SAFIRE despite the half dose^23^.

In the present study, we examined for the first time the effect of a combination of low tube voltage and iterative image reconstruction of the latest generation (SAFIRE) on HCC detection in a homogeneous patient population using a uniform gold standard.

**Figure 2:**
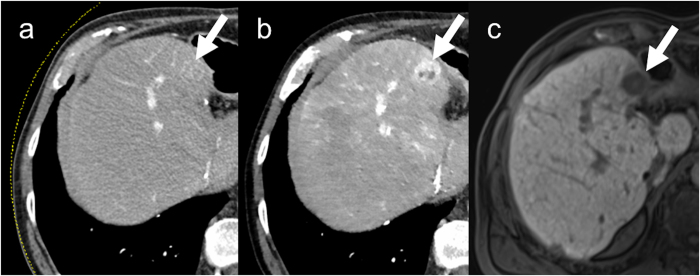
HCC detected on MRI in hepatobiliary phase (**c**). The arrow shows the HCC. (**a**) Barely visible at a tube voltage of 120 kV. (**b**) Effectively visualized at 80 kV with iterative image reconstruction.

We were able to show that the sensitivity for the detection of HCC was significantly higher with a low tube voltage using SAFIRE at iterative reconstruction settings I3 and I5 than with a high tube voltage using FBP (Fig. 2). The low tube protocol in combination with both iterative reconstruction strengths achieved an excellent sensitivity of 100% in detection of HCC lesions ≥3 cm, which is of high clinical relevance especially regarding potential liver transplantation according to the MILAN-criteria. In addition, the confidence level, i.e. the subjective confidence of the readers regarding the presence of HCC lesions, was significantly higher at a low tube voltage using iterative reconstruction. However, the number of false-positive results was comparable in all examined groups (80 kV IR3, 80 kV IR5 and 120 kV FBP).

The limitations of our study are the low patient number, the lack of histological evidence, albeit with definitive MRI results or in inconclusive cases combined with CEUS, and the unicentric setup.

Despite the limitations, we are convinced that the combination of iterative reconstructions and low tube voltage is superior to conventional protocols for the detection of HCC. Additional studies with histological examination of all, especially small HCC lesions after liver transplantation or surgical resection should be conducted to prove and emphasize this thesis.
